# Determinants and adverse perinatal outcomes of low birth weight newborns delivered in Hawassa University Comprehensive Specialized Hospital, Ethiopia: a cohort study

**DOI:** 10.1186/s13104-019-4155-x

**Published:** 2019-03-04

**Authors:** Melaku Desta, Mesfin Tadese, Bekalu Kassie, Mihrete Gedefaw

**Affiliations:** 1 Department of Midwifery, College of Medicine and Health Science, Debre Markos University, P.O. Box 226, Debre Markos, Ethiopia; 2 Department of Midwifery, College of Medicine and Health Science, Debre Berhan University, Debre Berhan, Amhara Region, Ethiopia; 3 Department of Nursing, College of Medicine and Health Science, Debre Markos University, Debre Markos, Ethiopia

**Keywords:** Low birth weight, Determinants, Perinatal outcome, Hawassa, Ethiopia

## Abstract

**Objective:**

Globally an estimated 15% to 20% of all births are low birth weight, representing more than 20 million births a year. Low birth weights are at a greater risk of both short and long-term sequels. Therefore, this study was conducted to assess determinants and perinatal outcomes of low birth weight newborns delivered in Hawassa University Comprehensive Specialized Hospital, Southern Ethiopia.

**Results:**

A total of 420 mothers were included in the study with a response rate of 97%. The mean birth weights of the neonate were 3360 (± 870 SD) grams and the incidence of low birth weight was found to be 16.6% (95% CI 13.46–18.38). Previous abortion [RR = 1.87 (2.53, 12.5)], hypertensive disorder [RR = 4.59 (4.93, 42.7)], having < 4 antenatal visits [RR = 3.45 (2.35, 13.8)] and prematurity [RR = 18.2 (6.24, 34.5)] was increased the risk of low birth weight. Low birth weight neonates were associated with a low Apgar score [RR = 18.2 (6.24, 34.5)] and early neonatal death [RR = 18.2 (6.24, 34.5)]. For this, identifying populations at the greatest risk of previous abortion, hypertensive disorders of pregnancy and prematurity were the major priorities aimed at reducing low birth weight. Incorporate mental health in the prenatal visit, improving the care for a high-risk pregnant woman was also recommended.

**Electronic supplementary material:**

The online version of this article (10.1186/s13104-019-4155-x) contains supplementary material, which is available to authorized users.

## Introduction

According to the World Health Organization (WHO), low birth weight is defined as a weight of less than 2500 g (5.5 lb) at birth. Low birth weight also includes preterm neonates, small for gestational age neonates at term and the combination of these two situations, who particularly have the worst perinatal outcomes [1]. Globally an estimated 15% to 20% of all births are low birth weight, representing more than 20 million births a year [1, 2].

The mother’s own fetal growth and her diet during adolescent and her body composition at conception are commonly associated with low birth weight. Other risk factors for low birth weight includes multiple gestations, previous abortion, young women, socio-economic status, infections, maternal nutrition and lifestyle, and medical disorders during pregnancy including hypertensive disorders, fetal infection, and anomalies and placental pathologic conditions [2–5].

Low birth weight is related to a range of both short and long-term sequels such as prematurity. Each year, about 1.1 million babies die from complications of prematurity. Low birth weight neonates have a > 20 times greater risk of dying than neonates with birth weight of > 2500 g [6, 7]. Those who survive tend to remain undernourished, with reduced muscle strength and growth, and have impaired immune function and this high risk of disease. Low birth weight neonates are not only at high risk of death but also are at increased risk of long-term neurologic disability, impaired language development, reduced cognitive abilities and IQ, and increased risk of medical disorders including cardiovascular disease and diabetes later in life [1, 8, 9]. Furthermore, due to the immaturity of multiple organs systems, a high risk of respiratory distress, interventricular hemorrhage, sepsis, blindness, and gastrointestinal disorders [10].

Currently, WHO and other organizations are working against reducing low birth weight by 30% by the year 2025 [1]. Despite those activities, low birth weight is increasing in Ethiopia based on Ethiopian Demographic and Health Survey report; 14% in 2005, 11% in 2011, and 13% in 2016 [11–13]. Concerning the perinatal outcomes, there is a paucity of study across the country. Therefore, this study aimed to assess the determinants and adverse perinatal outcomes of low birth weight newborns delivered in Hawassa University Comprehensive Specialized Hospital (HUCSH).

## Main text

### Study setting, design and period

A hospital-based retrospective cohort study design was conducted in Hawassa University Comprehensive Specialized Hospital (HUCSH) from July 10 to August 15, 2018. The hospital is found at Hawassa City, capital of Southern Nations Nationalities and People’s Region (SNNPR), which is 275 km far from the capital city of the country, Addis Ababa. HUCSH is one of the largest Hospital in the region, which serve as a specialized and teaching hospital at the regional level and provides both delivery and neonatal intensive care unit service. The hospital serves for more than 3 million populations for the region and two zones of Oromia region.

### Study population

All women who delivered in the hospital in the last 1 year were the source populations. There are 4056 women who gave birth in 1 year. Exposed groups were those newborns who had low birth weight (LBW) (< 2500 g) and non exposed or normal birth weight (NBW) was that newborn who weights greater than or equals to 2500 g and those delivered in the hospital on the same day as enrolled regardless of the mode of delivery and fetal outcome. All women who gave singleton after 28 weeks of gestation or weight of at least 1000 g were included. However, mothers with congenital anomalies like (hydrocephalus) and multiple pregnancies and incomplete data were excluded. LBW and NBW were selected after reviewing of women’s chart, delivery, and neonatal logbook.

### Sample size and sampling procedure

The sample size was determined using double population proportion formula using Open Epi version 3 software, using the following statistical assumptions, 80% power of the study, 95% confidence interval, case to control a ratio of 1 to 5 (r = 5) and considering prematurity and abruption as predictors of low birth weight and prevalence of stillbirth 1% among non exposed group with an odds ratio of 9 from a cohort study in Zambia [14] *and* making the final sample size of 434 (73 exposed and 361 unexposed). Exposed groups were selected using random sampling technique. For each exposed, five consecutive nonexposed newborns delivered in the hospital as soon as the exposed diagnosed on the same day in the same as of exposed will be selected as a control group.

### Data collection tool and measurement

Data were collected using structured and pre-tested questionnaire. The questionnaires were adapted from Ethiopian demographic health survey and other related literature [11–13]. Then the adapted questionnaires were modified and conceptualized to fit the research objectives. Admission history, labour follow up sheet, delivery summary and the antenatal care follow-up sheets from the mother’s or newborns obstetric records were reviewed to obtain the required information. The principal investigator examined the completeness, consistency, and accuracy of the collected data on regular bases. To assess the perinatal outcomes, the neonates were followed till admitted to the neonatal intensive care unit (NICU) or discharge to home (Additional file 1).

### Data processing and analysis

The data were coded, cleared and entered on Epi data 3.1 software and exported to Statistical package for social science (SPSS) software version 20 for analysis. Summary statistics such as mean and standard deviation and the proportion of the characteristics of LBW and NBW was computed using Chi square. Multivariable logistic regression was carried out to examine the association of the outcome variables with selected determinant factors. Variables that will be associated in bivariate logistic regression with the significance level of p-value < 0.25 entered into a multivariable logistic regression model to control possible confounding effects. Model was fitted (p-value = 0.39) using Hosmer and Lemeshow fit statistic. Variables with a p value < 0.05 will be considered as statistically significant factors and odds ratio with 95% confidence interval was used to measure the strength of association. The analysis was done by reporting guidelines of the Strengthening the Reporting of Observational Studies in Epidemiology (STROBE) statement checklist [15].

### Ethical considerations

Ethical clearance was obtained from Hawassa University, College of Medicine and Health Science, Institutional Review Board Ethical review committee with a Ref. No *IRB/163/10*. Then permission letter was taken from the Department of midwifery and Medical clinical director. A brief explanation was given on the objectives as well as the benefit of the study to the concerned officials. Confidentiality and privacy of every patient’s information were ensured.

## Result

### Socio-demographic and obstetric characteristics of the respondent

A total of 420 singleton births were included in this analysis with a response rate of 97%. The mean age the participant was 26.9 (± 5.5 SD) years and the birth weight was 3.36 kg (± 0.87). Seventy women had given a low birth weight (LBW) newborn, this constitutes an incidence of 16.6% (95% CI 13.46–18.38). Of those women who had LBW, 41 (58.6%) were in the age group of 20–34 years and above one third (68%) resides outside Hawassa. Mothers with LBW infants were more likely to be multigravida (61%), had a complication in the previous pregnancy (48.8%) and recent pregnancy (65.7%). Hypertensive disorder of pregnancy (37.1%) and antepartum hemorrhage (22.9%) were common among mothers gave birth with LBW newborn. Similarly, 33% of women with LBW had Antenatal care (ANC) visit; of that 60.4% had ANC visits < 4 times. Significant proportions (72.9%) of LBW were born preterm and nearly two-thirds (64.6%) of normal birth weight (NBW) newborns were male (Table 1).

**Table 1:** Sociodemographic and obstetrical characteristics of LBW in HUCSH, 2018

Variables	Category	LBW n (%)	NBW n (%)
Age	< 20	15 (21.4%)	15 (15.7%)
	20–34	41 (58.6%)	243 (69.4%)
	≥ 35	14 (20%)	52 (14.9)
Residence	Outside Hawassa	48 (68.6%)	221 (63.1%)
	Hawassa and around	22 (31.4%)	129 (36.9%)
Gravidity	One	27 (38.6%)	105 (30%)
	2–4	30 (42.9%)	149 (42.6%)
	≥ 5	13 (18.5%)	96 (27.4%)
Complication in previous pregnancy	Yes	21 (48.8%)	146 (59.6%)
	No	22 (51.2%)	99 (40.4%)
Previous abortion	Yes	9 (20.9%)	31 (12.7%)
	No	34 (79.1%)	217 (87.3%)
Complication in recent pregnancy	Yes	46 (65.7%)	100 (28.6%)
	No	24 (34.3%)	250 (71.4%)
Hypertensive disorder	Yes	26 (37.1%)	38 (50%)
	No	44 (62.9%)	38 (50%)
APH	Yes	16 (22.9%)	28 (36.8%)
	No	54 (87.1%)	48 (63.2%)
Infection	Yes	14 (20%)	13 (17.1%)
	No	56 (80%)	63 (82.9%)
Antenatal anemia	Yes	13 (18.6%)	7 (9.2%)
	No	60 (81.4%)	76 (90.8%)
ANC visit	Yes	16 (23%)	32 (9%)
	No	54 (77%)	318 (91%)
Frequency of ANC visit	< 4 visit	29 (60.4%)	115 (45.8%)
	≥ 4 visit	19 (39.6%)	136 (54.2%)
Gestational age	< 37 week	51 (72.9%)	32 (9.1%)
	> 42 week	4 (5.7)	69 (19.7%)
	37–42 week	15 (21.4%)	249 (71. 2%)
Sex of the fetus	Male	36 (51.4%)	226 (64.6%)
	Female	34 (48.6%)	124 (35.4%)

### Determinants of low birth weight

In bivariate analysis, 11 variables were significant and fitted for multivariable regression with p-value < 0.25. Only 4 variables; previous abortion, a hypertensive disorder of pregnancy, the frequency of ANC visit and gestational age at birth remained determinants of LBW (Table 2). Those women who have a history of previous abortion in their lifetime [RR = 1.87 (2.53, 12.5)] had two times the risk of LBW than those who haven’t the event. Women who had a hypertensive disorder of pregnancy [RR = 4.59 (4.93, 42.7)] had 4 and half times risk to gave LBW newborn than their counterparts. Similarly, the risk of LBW was higher among women who have < 4 ANC visits [RR = 3.45 (2.35, 13.8)]. Preterm birth was the strongest determinant for LBW [RR = 18.2 (6.24, 34.5)], premature newborns were 18 times more likely to be LBW.

**Table 2:** Determinants of Low birth weight in HUCSH, Southern Ethiopia, 2018

Variables	Category	LBW	NBW	CRR [95% CI]	ARR [95% CI]
Age	< 20	15	15	1.01 (0.45, 2.33)	1.56 (0.76, 3.84)
	20–34	41	243	0.63 (0.32, 1.23)	0.85 (0.45, 2.95)
	≥ 35	14	52	1	1
Gravidity	One	27	105	1.89 (0.97, 4.67)	2.33 (0.41, 12.3)
	2–4	30	149	1.64 (0.81, 3.38)	2.01 (0.03, 2.43)
	≥ 5	13	96	1	1
Complication in previous pregnancy	Yes	21	146	0.65 (0.34, 1.24)	0.87 (0.06, 12.6)
	No	22	99	1	1
Previous abortion	Yes	9	31	1.83 (0.83, 4.38)	1.87 (2.53, 12.5)
	No	34	217	1	1
Complication in recent pregnancy	Yes	46	100	4.95 (2,85, 8.61)	3.43 (0.65, 4.78)
	No	24	250	1	1
Hypertensive disorder	Yes	26	38	4.91 (2.71, 8.89)	4.59 (4.93, 42.7)
	No	44	38	1	1
APH	Yes	16	28	3.45 (1.75, 6.87)	2.39 (0.21, 25.5)
	No	54	48	1	1
Infection	Yes	14	13	6.89 (3.03, 15.7)	4.00 (0.26, 60.3)
	No	56	63	1	1
Antenatal anemia	Yes	13	7	2.35 (0.93, 7.13)	0.18 (0.01, 3.32)
	No	60	76	1	1
Frequency of ANC visit Gestational age	< 4 visit	29	115	1.59 (0.85, 2.98)	3.45 (2.35, 13.8)
	≥ 4 visit	19	136	1	1
	< 37 week	51	32	23.5 (11.9, 46.3)	18.2 (6.24, 34.5)
	> 42 week	4	69	0.87 (0.28, 2.71)	0.57 (0.03, 14.4)
	37–42 week	15	249	1	1
Sex of the fetus	Male	36	226	0.56 (0.33, 0.94)	0.45 (0.23, 2.48)
	Female	34	124	1	1

### Perinatal outcomes of low birth weight

Above two-thirds (68%) of the LBW neonates and nearly half (48%) of the NBW neonates had at least one adverse perinatal outcomes. LBW newborns were at the higher risk of Low Apgar score (42%), early neonatal death (19.3%) and NICU admission (22.6%). LBW neonates were two times more likely at risk of low Apgar score [RR = 2.29 (1.88, 5.96)] than the NBW group. The risk of early neonatal death was three times [RR = 3.02 (1.63, 6.26)] more likely among the LBW newborns than the NBW neonates (Table 3).

**Table 3:** Perinatal outcomes of low birth weight in HUCSH, Southern Ethiopia, 2018

Perinatal outcomes	Category	Birth weight (Kg)		CRR [95% CI]	ARR [95% CI]
		LBW	NBW		
Perinatal complications	Yes	48 (68.9%)	168 (48%)	2.65 (1.51, 4.64)*	2.87 (0.34, 24.5)
	No	22 (31.1%)	182 (52%)	1	1
Still birth (n = 216)	Yes	17 (35.4%)	50 (30%)	1.02 (0.58, 1.79)	
	No	31 (64.6%)	118 (70%)	1	
Low Apgar (n = 149)	Yes	13 (42%)	23 (19.5%)	3.33 (1.93, 5.77)*	2.29 (1.88, 5.96)**
	No	18 (58%)	95 (80.5)	1	1
Early neonatal death	Yes	6 (19.3%)	8 (6.7%)	3.34 (1.28, 8.66)	3.02 (1.63, 6.26)**
	No	25 (80.7%)	108 (93.3)	1	1
NICU admission	Yes	7 (22.6%)	12 (10%)	2.34 (1.17, 4.65)*	2.52 (0.19, 3.26)
	No	24 (87.4%)	106 (90%)	1	1

*Kg* implies weight in kilogram

* showed variables fitted for mutivariable logistic regresion

** shows significant at p-value < 0.05

## Discussion

The study aimed to assess the determinants and perinatal outcomes of low birth weight at Hawassa specialized Hospital, Southern Ethiopia. It was found that mothers who have a previous abortion, the frequency of ANC visit; gestational age at birth and hypertensive disorders of pregnancy was statistically significant determinants of low weight at birth. The incidence of LBW in this hospital is 16.6%, which is in line with a country findings of the recent meta-analysis, 17% [16]. This is higher than the findings of Zambia [14] and Tanzania [17], showed that 10.6% of LBW. This might be due to a high prevalence of home delivery, preterm delivery, hypertension during pregnancy, antepartum hemorrhage, and study area difference, in our case tertiary hospital, increased the referral of complicated cases, increased risk of LBW.

The study revealed that those mothers who have at least one previous abortion were two times more likely to deliver LBW neonates compared to those who have no abortion history. This is similar to studies conducted in Denmark and the US [18, 19], and a meta-analysis was done in Canada [20]. The study also supported by Bossley [21] found that women who had an abortion in the first or second trimester had a 35% increased risk of a LBW baby and a 36% raised risk of a pre-term baby in later pregnancies and Tsegaye et al. [22] revealed that previous adverse pregnancy outcomes were associated with the recent outcome. The possible explanation for this might be most likely to be physical damage to the cervix caused methods of abortion, reduce the tensile strength of the cervical plug, result in the preterm birth subsequently LBW and due to psychological stress for the previous occurrence of the event. Hence stress-depression is associated with previous abortion [23] and other pregnancy complications [24] and lowers dietary diversity of women [25, 26], reduces fetal nutrients vital for development leads to an increased risk of LBW [27–30]. Conversely, various kinds of literature suggested that induced abortion does not increase the risk for low birth weight in the subsequent pregnancy [31–33]. This might be explained by the differences in the methods used to perform abortion at different times and different countries.

This study found that one of the major causes of adverse pregnancy outcomes, the hypertensive disorder of pregnancy has a significant impact on pregnant women to have LBW neonate. There was greater risk of delivering a low weight infant among mothers with hypertension during pregnancy as compared to those who did not develop the disease. The effect is more pronounced if delivery occurs before reaching term [34]. Similar studies shown comparable results [17, 35–40], suggested that the significant association of hypertension disorder and LBW. This explained by the fact that hypertension cause uteroplacental insufficiency. Similarly, the study revealed that mothers who had less than four ANC visit were more at risk to deliver a low weight neonate compared to mothers who attended more than four times. This is comparable to similar studies [35, 41–44]. This is also supported with studies done by Gizaw et al. [28], Mahmud et al. [41], Oulay et al. [45] and Kamala et al. [46] revealed that ANC visit < 4 times were associated with LBW.

In addition to this, premature birth was another most significant determinant of LBW. Those women who deliver before 37 completed weeks are more at risk to give low weight births than those giving birth at term. This is in accordance with different studies [35, 36, 42, 47–51] and a meta-analysis did in Ethiopia [16] stated that delivery remote from term associated with LBW. When the neonates delivered before reaching term, they are likely to be small and the babies are more likely to have decreased skeletal muscle mass and subcutaneous fat tissue [52].

Likewise, the study found that LBW newborns are associated with an increased risk of a low Apgar score and early neonatal death. The finding is in line with a study in Brazil [50] and Tanzania [17] have found that LBW infants had a higher risk of having Apgar scores below 7 at the 5th min and Yasmin in Bangladesh [53] had ascertained the role of LBW on the increased risk of early neonatal death. Similarly, the finding is in accordance with studies done by Bayou [40], Sangamam [54], Chibwesha [14] and Mitao [17] demonstrated that early neonatal mortality rate was higher among LBW babies. These facts indicate that low weight at birth increases the risk of intrauterine growth restriction and early neonatal death. It also explained due to the high burden of preterm birth (73%) and its complications, which is expected to have a higher risk for mortality. Hence, several organ systems of human fetus usually immature before the end of 37 weeks of gestational age leads to difficult to maintain the extra uterine environment, particularly from pulmonary hypoplasia, end up with mortality.

## Conclusion

Low birth weight neonates are still at increased risk of adverse perinatal outcomes in the study area. Identifying populations at greatest risk of previous abortion and incorporate mental health in the prenatal visit, hypertensive disorders of pregnancy and prematurity were the major priorities and fundamental strategies for the success of programmers and policies aimed at reducing low birth neonates. The early screening of high-risk pregnancy and the care provided for low birth weight newborn should be improved and further prospective study such as on the type of LBW (very LBW and Extremely very LBW) on perinatal outcomes should be addressed.

## Limitations

Despite it was a cohort study and extensive efforts have been made, the finding could be interpreted in the presence of some inevitable limitations. The study might underestimate early neonatal death due unable to assess outcome after NICU admission and death after discharge and its retrospective nature might prevent some variables, as educational level, Iron-folic acid supplementation, maternal mental health and dietary pattern of women.

## Additional file

**Additional file 1.** English version Questionnaire of determinants and outcomes of low birth weight in HUCSH, Southern Ethiopia, 2018.

## Abbreviations

ANC: Ante Natal Care
ARR: adjusted relative risk
HUCSH: Hawassa University Comprehensive Specialized Hospital
LBW: low birth weight
NBW: normal birth weight
NICU: neonatal intensive care unit
SNNPR: Southern Nations Nationalities and People’s Region
STROBE: Strengthening the Reporting of Observational Studies in Epidemiology
WHO: World Health Organization
