# Supplementary material for: Determinants and adverse perinatal outcomes of low birth weight newborns delivered in Hawassa University Comprehensive Specialized Hospital, Ethiopia: a cohort study
Source: BMC Res Notes. 2019 Mar 4;12:118. doi: 10.1186/s13104-019-4155-x (PMC6399950; doi:10.1186/s13104-019-4155-x)
Supplement: Supplementary file 1 — Additional file 1. English version Questionnaire of determinants and outcomes of low birth weight in HUCSH, Southern Ethiopia, 2018. [file 13104_2019_4155_MOESM1_ESM.docx]

**Additional file:** English version Questionnaire of determinants and outcomes of low birth weight in HUCSH, Southern Ethiopia, 2018

| **Variables** | **Response** | **Skip** |
| --- | --- | --- |
| **100. Characteristics of LBW** |  |  |
| 101. Birth weight in gram | - |  |
| 102. Maternal age (years) |  |  |
| 103. Residence | 1. Outside Hawassa  2. Hawassa & around |  |
| 104. Gravidity | - |  |
| 105. Complication in previous pregnancy, if multi gravida | 1. Yes 2. No | If 2, skip to Q 106 |
| 106. What complications encountered? | - |  |
| 107. Did the women has ANC visit? | 1. Yes 2. No | If 2, skip to Q 108 |
| 108. How many visit did she followed? | 1. < 4 visits 2. ≥ 4 visits |  |
| 109. What is the gestational age in weeks? | - |  |
| 110. What was the sex of newborn | 1. Male 2. Female |  |
| **200. perinatal outcomes of LBW** | **Response** | **Skip** |
| 201. Was any perinatal complication? | 1. yes 2. No | If 2 completed |
| 202. Was stillbirth? | 1. yes 2. No |  |
| 203. Was the APGAR score is low? | 1. yes 2. No |  |
| 204. Was are any Early neonatal death? | 1. yes 2. No |  |
| 205. NICU admission? | 1. yes 2. No |  |
